# The Biallelic Inheritance of Two Novel *SCN1A* Variants Results in Developmental and Epileptic Encephalopathy Responsive to Levetiracetam

**DOI:** 10.3390/biomedicines12081698

**Published:** 2024-07-31

**Authors:** Giorgia Dinoi, Elena Conte, Orazio Palumbo, Mario Benvenuto, Maria Antonietta Coppola, Pietro Palumbo, Patrizia Lastella, Brigida Boccanegra, Ester Di Muro, Marco Castori, Massimo Carella, Vittorio Sciruicchio, Marina de Tommaso, Antonella Liantonio, Annamaria De Luca, Angela La Neve, Paola Imbrici

**Affiliations:** 1 Department of Pharmacy—Drug Sciences, University of Bari “Aldo Moro”, 70125 Bari, Italy; giorgia.dinoi@uniba.it (G.D.); elena.conte@uniba.it (E.C.); maria.coppola@uniba.it (M.A.C.); brigida.boccanegra@uniba.it (B.B.); antonella.liantonio@uniba.it (A.L.); annamaria.deluca@uniba.it (A.D.L.); 2 Division of Medical Genetics, Fondazione IRCCS-Casa Sollievo della Sofferenza, 71013 San Giovanni Rotondo, Italy; o.palumbo@operapadrepio.it (O.P.); m.benvenuto@operapadrepio.it (M.B.); p.palumbo@operapadrepio.it (P.P.); e.dimuro@operapadrepio.it (E.D.M.); m.castori@operapadrepio.it (M.C.); m.carella@operapadrepio.it (M.C.); 3 Centro Sovraziendale Malattie Rare, UOC Medicina Interna Universitaria “C. Frugoni”, AOU Policlinico Consorziale di Bari, 70124 Bari, Italy; patrizia.lastella@uniba.it; 4 Children Epilepsy and EEG Center, Ospedale San Paolo di Bari, 70123 Bari, Italy; vittorio.sciruicchio@asl.bari.it; 5 DiBraiN Department, University of Bari “Aldo Moro”, 70124 Bari, Italy; marina.detommaso@uniba.it

**Keywords:** *SCN1A*, Nav1.1, epilepsy, patch-clamp, biallelic inheritance

## Abstract

Loss-, gain-of-function and mixed variants in *SCN1A* (Nav1.1 voltage-gated sodium channel) have been associated with a spectrum of neurologic disorders with different severity and drug-responsiveness. Most *SCN1A* variants are heterozygous changes occurring de novo or dominantly inherited; recessive inheritance has been reported in a few cases. Here, we report a family in which the biallelic inheritance of two novel *SCN1A* variants, N935Y and H1393Q, occurs in two siblings presenting with drug-responsive developmental and epileptic encephalopathy and born to heterozygous asymptomatic parents. To assess the genotype–phenotype correlation and support the treatment choice, HEK 293 cells were transfected with different combinations of the *SCN1A* WT and mutant cDNAs, and the resulting sodium currents were recorded through whole-cell patch-clamp. Functional studies showed that the N935Y and H1393Q channels and their combinations with the WT (WT + N935Y and WT + H1393Q) had current densities and biophysical properties comparable with those of their respective control conditions. This explains the asymptomatic condition of the probands’ parents. The co-expression of the N935Y + H1393Q channels, mimicking the recessive inheritance of the two variants in siblings, showed ~20% reduced current amplitude compared with WT and with parental channels. This mild loss of Nav1.1 function may contribute in part to the disease pathogenesis, although other mechanisms may be involved.

## 1. Introduction

The *SCN1A* gene encodes for Nav1.1, one of the four voltage-gated sodium channels alpha subunits that contribute to the initiation and propagation of action potentials in the brain [1]. Both loss-, gain-of-function (LoF, GoF) and mixed variants in *SCNA1* have been identified in patients associated with a wide spectrum of neurologic disorders, including epilepsy and neurodevelopmental disorders with different severities and drug-responses [2,3]. *SCN1A* LoF variants include Dravet syndrome, a severe drug-resistant developmental and epileptic encephalopathy in which sodium channel blockers are contraindicated, and genetic epilepsy with febrile seizures plus (GEFS+), a milder drug-responsive epilepsy form [4]. GoF *SCN1A* variants have been traditionally associated with familial hemiplegic migraine type 3 (FHM3). Recently, GoF variants have also been identified in severe neonatal or early infantile developmental and epileptic encephalopathy, with or without movement disorder and arthrogryposis, that, in most cases, respond to sodium channel blockers [2,5,6]. In-between, variants with mixed GoF/LoF phenotypes have also been found to be associated with early infantile developmental and epileptic encephalopathy with neonatal seizures, profound developmental impairment and hyperkinesia [2,7]. Due to the identification of both GoF and LoF variants with diverse severity, modes of inheritance and drug responsiveness, deciphering the disease mechanisms of novel variants through functional studies represents the first step to understanding pathogenicity. This helps guide the choice of treatments that target the functional consequences of the mutation and allows avoiding contraindicated antiseizure medications [8].

Most *SCN1A* pathogenic variants are located at conserved positions in the channel structure. They occur as heterozygous changes dominantly inherited or appearing de novo. Recessive inheritance has also been reported in eight families in which 12 affected children harbor homozygous or compound heterozygous *SCN1A* missense variants, whereas their heterozygous parents are asymptomatic [9,10,11,12,13,14] (Table 1). No functional study was provided for any of the homozygous or compound heterozygous variants reported in the literature; thus, the molecular mechanisms underlying the clinical phenotypes remained speculative.

Here, we provide the clinical and genetic evidence of four family members carrying two novel *SCN1A* variants, c.2803A>T, p.(Asn935Tyr) (named N935Y in the paper) (paternal origin) and c.4179T>A, p.(His1393Gln) (named H1393Q in the paper) (maternal origin), involving two non-conserved amino acids located in the extracellular loops of the Nav1.1 channel. The heterozygous parents are asymptomatic, whereas their two children (male and female), who show biallelic inheritance of both variants, have developmental and epileptic encephalopathy characterized by drug-responsive familial myoclonic epilepsy, cognitive delay and postural abnormalities. To gain insight into the pathogenicity of the genotype in the family and to attempt to decipher the molecular mechanism underlying the disease when both variants were inherited, we assessed the functional behavior of the two novel Nav1.1 mutations, H1393Q and N935Y, alone and in combination in a heterologous expression system.

## 2. Materials and Methods

### 2.1. Clinical and Genetic Analysis

Patients were evaluated at the DiBraiN Department of the University of Bari “Aldo Moro” and at the Division of Medical Genetics, Fondazione IRCCS-Casa Sollievo della Sofferenza, San Giovanni Rotondo (Foggia) in Italy in 2021 and evaluated for a possible genetic etiology of their clinical picture.

Peripheral blood samples were collected from both the probands and their parents, and genomic DNA was isolated using Bio Robot EZ1 (Qiagen, Solna, Sweden). The quality of DNA was tested on a 1% electrophoresis agarose gel, and the concentration was quantified using a Nanodrop 2000C spectrophotometer (Thermo Fisher Scientific, Waltham, MA, USA).

The probands’ DNA was analyzed by targeted resequencing (TRS) using a SureSelect gene panel (Agilent Technologies, Boulder, CO, USA) designed to selectively capture 135 known genes associated with syndromic and non-syndromic forms of epilepsy. Libraries were prepared using the SureSelect target enrichment kit (Agilent Technologies, Boulder, CO, USA) following the manufacturer’s instructions. Targeted fragments were then sequenced on a NextSeq 500 sequencer (Illumina, San Diego, CA, USA) using the NextSeq 500 mid-output kit V2.5 (300-cycle flow cell).

Reads were aligned to the GRCh37/hg19 reference genome by Burrows–Wheeler Aligner (BWA) (v.0.7.17). BAM files were sorted using SAMtools (v.1.7) and purged from duplicates using the Mark Duplicates tool from the Picard suite (v.2.9.0). Mapped reads were locally realigned using GATK 3.8. Reads with mapping quality scores lower than 20 or with more than one-half nucleotides with quality scores less than 30 were filtered out. The GATK’s HaplotypeCaller tool was used to identify variants [15], which were annotated based on frequency, impact on the encoded protein, conservation and expression using distinct tools, as appropriate (ANNOVAR, dbSNP, 1000 Genomes, EVS, GnomAD, ESP, KAVIAR and ClinVar) [16,17,18,19,20]. Precomputed pathogenicity predictions were retrieved using dbNSFP v 3.0 (PolyPhen-2, SIFT, MutationAssessor, FATHMM, LRT and CADD) [21], as well as evolutionary conservation measures. Next, variant prioritization was performed. Firstly, variants described as benign and likely benign were excluded. Then, remaining variants were classified based on their clinical relevance as pathogenic, likely pathogenic, or variant of uncertain significance according to following criteria: (i) nonsense/frameshift variant in genes previously described as disease-causing by haploinsufficiency or loss-of-function; (ii) missense variant located in a critical or functional domain; (iii) variant affecting canonical splicing sites (i.e., −1 or −2 positions); (iv) variant absent in allele frequency population databases; (v) variant reported in allele frequency population databases, but with a minor allele frequency (MAF) significantly lower than expected for the disease (<0.002 for autosomal recessive disease and <0.00001 for autosomal dominant disease); and (vi) variant predicted and/or annotated as pathogenic/deleterious in ClinVar and/or LOVD. The resulting putative pathogenic variants were confirmed by Sanger sequencing of both the probands’ and the parents’ DNA. PCR products were sequenced using the BigDye Terminator v1.1 Sequencing Kit (Applied Biosystems, Foster City, CA, USA) and the ABI Prism 3100 Genetic Analyzer (Thermo Fisher Scientific, Waltham, MA, USA). The clinical significance of the identified putative variants was interpreted according to the American College of Medical Genetics and Genomics (ACMG) guidelines [22]. The variant analysis was carried out considering the ethnicity of the patients. Nucleotide variant nomenclature follows the format indicated in the Human Genome Variation Society (HGVS, http://www.hgvs.org) recommendations (accessed on 2 June 2023).

### 2.2. Mutagenesis and Nav1.1 Channel Expression

Mutations were introduced into the plasmid pCDNA3-hNav1.1 (generously gifted by Dr. Michael Pusch; [23]) using the QuickchangeTM site-directed mutagenesis kit (Agilent, Santa Clara, CA, USA). HEK 293 cells were transiently transfected in 100 mm dishes with WT (7 μg) or mutant (7 μg) pCDNA3-hNav1.1 and the CD8 receptor gene reporter, using the calcium phosphate precipitation method [24]. The complete coding region of the cDNA was sequenced to exclude polymerase errors. The transfected cells were identified by microbeads coated with anti-CD8 antibodies (Dynabeads M-450 CD8; Dynal, Great Neck, NY, USA) and were used for electrophysiological recordings. For co-expression experiments, an equal amount of WT and mutant channel cDNAs (7 μg + 7 μg) was transfected, and the resulting currents were compared with those generated by transfecting Nav1.1 WT cDNA alone (14 μg).

### 2.3. Electrophysiology

Whole-cell sodium currents were recorded at room temperature from HEK 293 cells 48 h after transfection using an Axopatch 200A amplifier, a Digidata 1550B digitizer, and the pClamp 10.6 software (Molecular Devices, San Jose, CA, USA). Current signals were filtered at 5 kHz and sampled at 10 kHz. Patch-clamp pipettes were pulled from borosilicate glass using a vertical puller (Narishige, London, UK) to a resistance of 3–4 MΩ. The intracellular solution contained (in mM): NaCl, 10; CsF, 120; CsCl, 10; EGTA/CsOH, 5; and HEPES, 5 (pH 7.2 with CsOH). The extracellular solution contained (in mM): NaCl, 150; KCl, 4; CaCl_2_, 2; MgCl_2_, 1; glucose, 5; and HEPES, 5 (pH 7.4 with NaOH) [23,25].

Recordings were generally initiated 5 min after establishment of whole-cell configuration to allow a complete dialysis of the cytoplasm and stabilize the initial time-dependent shift in the voltage-dependence; the different protocols were run in the same conditions and at the same time points. Only cells with access resistance <7 MΩ were used. The capacitance currents and series resistances (80%) were partially compensated using the amplifier circuit. Leak currents were always <150 pA at −100 mV. Cells exhibiting peak current amplitudes >3500 pA were excluded from analyses. Patch-clamp recordings were analyzed off-line using ClampFit 10.6 (Molecular Devices, San Jose, CA, USA) and Prism–GraphPad (GraphPad Software (https://www.graphpad.com/), 225 Franklin Street, Boston, MA, USA).

To determine the current–voltage relationships, currents were elicited by 20 ms depolarizing pulses from −100 to +70 mV in 5 mV increments, from a holding potential of −150 mV (5 s interpulse duration). Peak current amplitudes at each voltage step were defined as the maximal current amplitudes. Current amplitudes were normalized to the cell capacitance (Cm) to obtain current density (pA/pF) [26].

The voltage-dependence of activation was measured using the same protocol. To analyze the voltage-dependence of activation, peak current amplitudes at each voltage were converted to conductance (G) as follows:

GNa = INa/(Vm − Erev),

where Vm is the voltage clamp step and Erev is the reversal potential for sodium ions in our experimental conditions. The values of GNa were normalized to the maximum conductance and plotted as a function of voltage. Conductance–voltage relationships were fitted with the Boltzmann equation:

G/Gmax = 1/(1 + exp (−(Vm − V1/2)/k)),

in which V1/2 is the membrane potential for half-maximum activation and k is the slope factor.

The voltage-dependence of steady-state inactivation was measured using a standard two-pulse protocol where a 100 ms conditioning prepulse to membrane potentials, between −150 and −20 mV (to induce steady-state inactivation) in 10 mV increments, was followed by a 20 ms test pulse to −20 mV, from a holding potential of −150 mV (5 s interpulse duration). Current amplitudes measured at each test pulse were normalized to the maximal current amplitude (Imax) and plotted as a function of prepulse voltage. The steady-state inactivation curves were fitted with the Boltzmann equation:

I/Imax = 1/(1 + exp ((Vm − V1/2)/k)),

in which V1/2 is the membrane potential for half-inactivation and k is the slope factor.

Sodium current decay at −10 mV was fitted with a double exponential function to calculate the inactivation time constants, using the equation:

I(t) = I0 + A1 × exp(−t/τfast) + A2 × exp(−t/τslow),

where A is the amplitude and τfast and τslow are the decay time constants.

Recovery from inactivation was assessed using a standard two-pulse protocol (5 s interpulse duration) to −20 mV from a holding potential of −150 mV. The voltage between the pulses was −150 mV, and the interpulse interval was varied between 0.3 and 1000 ms. Peak current amplitudes measured at each test pulse were normalized to current amplitudes measured during each prepulse and plotted against interpulse intervals. The time course of recovery from inactivation was analyzed by fitting the current amplitude ratio (Ifinal/Ipeak) with the single exponential function:

I(t) = [A × exp(t/τ)],

where τ is the recovery time constant and A is the amplitude.

### 2.4. Statistical Analysis

All data were reported as mean ± standard error of the mean (SEM) from the indicated number of cells. Multiple statistical comparisons between groups (WT, N935Y, H1393Q and WT + WT, WT + H1393Q, WT + N935Y and N935Y + H1393Q) were performed by a one-way analysis of variance (ANOVA), followed by the Bonferroni t-test post hoc correction when the null hypothesis was rejected (*p* < 0.05).

## 3. Results

### 3.1. Clinical Description

Patient II.1 is a 44-year-old woman with intellectual impairment (IQ = 49) and a history of epilepsy. The onset of epilepsy occurred at the age of 7 years, with generalized tonic-clonic seizures and seizures with impaired awareness. Around the age of 10 years and for some years later, patient II.1 presented sporadic episodes of impaired awareness and staring associated with the appearance of diffuse atypical spike and wave abnormalities at EEG. Seizures were treated, with partial success, with lamotrigine 400 mg/day replaced in 2015 by levetiracetam 2000 mg daily, resulting in complete seizure control.

Patient II.2 is a 38-year-old man with intellectual impairment (IQ = 50) and a history of epilepsy. The onset of epilepsy occurred at the age of 9 years, with generalized tonic-clonic seizures initially treated with carbamazepine. Under carbamazepine treatment, patient II.2 presented generalized convulsive status epilepticus. This drug was thus replaced in 2014 with levetiracetam 2000 mg/day, leading to the disappearance of the seizures. Since the age of 23, he has had lower limb “tremors”. Patient II.2 never presented episodes of impaired awareness and staring. Neither patient presented with fever sensitivity, while psychomotor delay was evident from the beginning.

In other facilities, both siblings had undergone karyotyping, measurement of urinary lactic acid and pyruvate levels, muscle biopsy, skin biopsy (negative for the accumulation of ceroid lipofuscin), search for NARP, MILS and MERRF mitochondrial DNA mutations in blood, genetic investigation for progressive myoclonic epilepsies, EMG (conduction velocity), jejunal biopsy for celiac disease and cystatin B gene analysis. All these investigations yielded normal findings.

In both patients, neurological examination conducted in our center revealed: slightly dysarthric speech, “jerky” slow eye movements, diffuse hypotonia, slight hesitancy in the index finger-to-nose and heel-to-knee tests; wide-based and slightly hesitant gait; impaired tandem gait; and moderate fragmentary myoclonus, both postural and during movement of the limbs, particularly the lower limbs. In both cases, polygraphic EEG recording showed a slight slowing of basal brain electrical activity and a few rapid, spike-like paroxysms over the vertex leads, with a tendency to spread. There was no sign of photosensitivity. Brain MRI (on a 3 Tesla system) gave normal findings in patient II.1, while patient II.2 showed a faint signal hyperintensity of the white matter adjacent to the ventricular trigones posterior to the medial cells in the position of the semioval centers bilaterally. Genetic counseling was recommended.

### 3.2. Genetic Analysis

**Figure 1:**
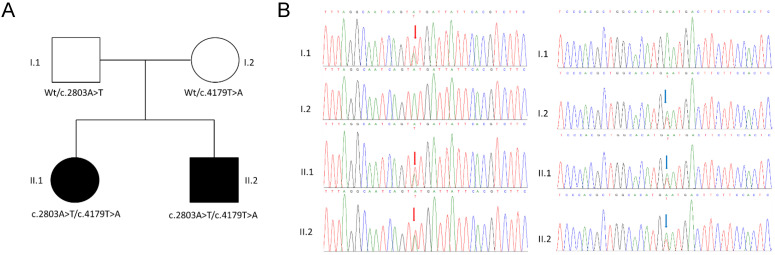
(**A**) Pedigree of the family displaying the compound heterozygous variant. Filled and unfilled circles/squares represent affected and unaffected individuals, respectively. (**B**) Electropherograms of DNA sequencing for the probands (II.1, II.2) and their parents (I.1, I.2). The variants c.2803A>T, p.(Asn935Tyr), inherited from the father, and c.4179T>A, p.(His1393Gln), inherited from the mother, identified here, are indicated by red and blue arrows, respectively.

The TRS approach allowed us to identify two heterozygous missense variants c.2803A>T, p.(Asn935Tyr) and c.4179T>A, p.(His1393Gln) in the *SCN1A* (NM_001165963.4) gene in the patients (patient II.1 and patient II.2). Both variants were novel and not found in gnomAD exomes and genomes (MAF = 0) and were classified as likely pathogenic according to the American College of Medical Genetics and Genomics guidelines. Co-segregation analysis was investigated in the family. The primers were designed using Primer3.0 (https://primer3.ut.ee/) (accessed on 2 June 2023) (*SCN1A*, exon 18, forward primer: TTAGCCATGAGCCTGAGACG; *SCN1A*, exon 18, reverse primer: GGTCCCAGCACATCCTTTTA; *SCN1A*, exon 24, forward primer: CTTTTAATGCTTCTCCCTCCCT; and *SCN1A*, exon 24, reverse primer: CCATCTGGGCTCATAAACTTGT) and the polymerase chain reaction (PCR) was performed under standard conditions. The PCR products (843 bp and 700 bp) were sequenced on an ABI 3500xL DNA Analyzer (Applied Biosystems, Foster City, CA, USA). The parental DNA analysis showed that c.2803A>T, p.(Asn935Tyr) was inherited from the father (parent I.1), while c.4179T>A, p.(His1393Gln) was inherited from the mother (parent I.2) (Figure 1).

### 3.3. Functional Characterization of Mutant Nav1.1 Currents

**Figure 2:**
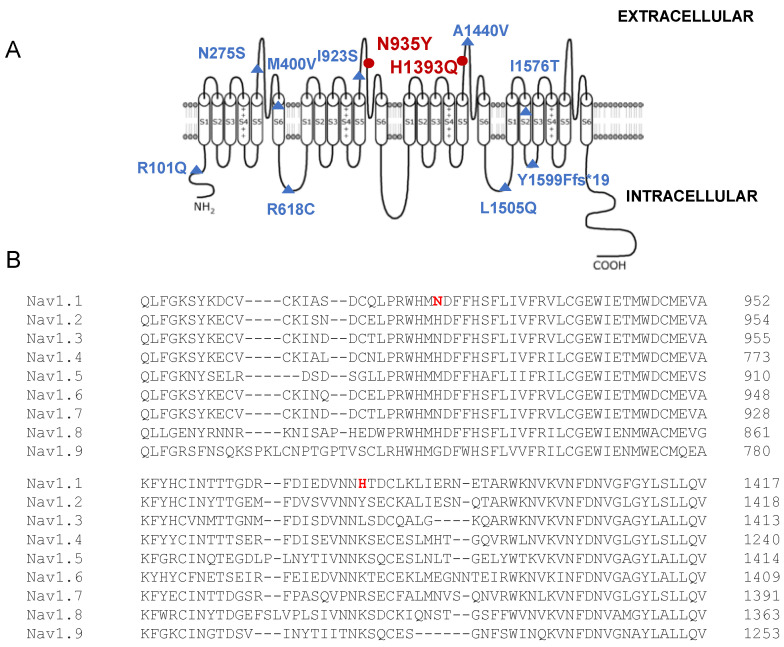
(**A**) Schematic representation of the Nav1.1 channel, showing the amino acidic substitutions involved in biallelic inheritance (Table 1). Red dots represent the identified mutations N935Y and H1393Q; and (**B**) Amino acid alignment of Nav1 channels.

The alpha subunit of Nav1.1 consists of four homologous domains (D1–4) each formed of six transmembrane segments (S1–S6), with the S1–S4 segments forming the voltage sensor domain (VSD), the S5-P-S6 segments forming the pore domain, and the IFM motif functioning as the inactivation particle. The H1393Q variant is located in the extracellular DIIIS5-P loop, whereas the N935Y is located in the DIIS5-P loop in the Nav1.1 structure (Figure 2A). Neither amino acid substitution affects a highly conserved position in the protein (Figure 2B).

**Figure 3:**
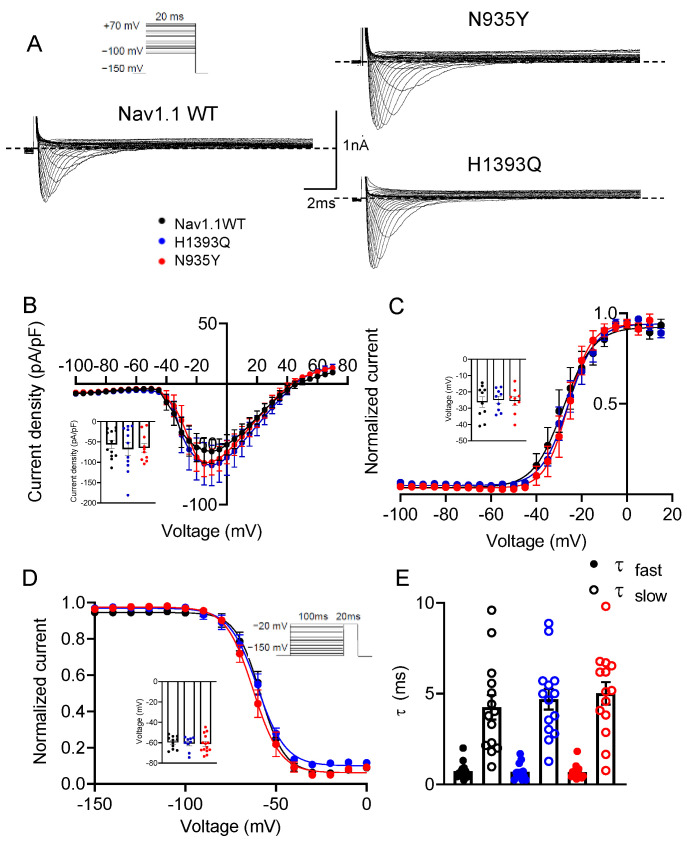
(**A**) Representative current traces evoked by 20 ms depolarizing steps from a holding potential of −150 mV to +70 mV from Nav1.1 WT (7 μg), N935Y (7 μg) and H1393Q (7 μg) channels expressed in HEK 293 cells. The voltage protocol is indicated in the upper panel in (**A**); (**B**) Current–voltage relationship for Nav1.1 WT, N935Y and H1393Q channels (n = 10–12). The inset shows current density measured at −10 mV for the three channel types; (**C**) Voltage-dependent activation curves for Nav1.1 WT, N935Y and H1393Q channels were obtained by plotting the normalized conductance as a function of the membrane potentials and fitting data points with a Boltzmann function (n = 9–10 cells). The inset shows V_1/2_ values for the four channel types; (**D**) Steady-state fast inactivation curves for Nav1.1 WT, N935Y and H1393Q channels were obtained by plotting the normalized peak tail currents measured at −20 mV as a function of the prepulse potentials and fitting data points with a Boltzmann function (n = 9–14 cells). The inset shows V_1/2_ values for the four channel types; and (**E**) Bar graphs showing the time constants of the fast inactivation measured at −10 mV for the indicated channels, calculated by fitting current decay with a double exponential function (n = 14 cells).

To verify the pathogenicity of the single variants compared with the WT, we first transfected HEK 293 cells with an equal amount of WT, H1393Q or N935Y cDNAs alone (7 μg), and recorded sodium currents through whole-cell patch-clamp (Figure 3A). As quantified in the relative IV curves, the H1393Q and N935Y channels showed sodium current densities comparable with those of Nav1.1 WT at −10 mV (−56.0 ± 9.9 pA/pF, −67.5 ± 16.9 pA/pF and −64.7 ± 10.8 pA/pF for WT, H1393Q and N935Y, respectively; Figure 3B; Table 2). Neither mutation affected the voltage-dependent activation, steady-state inactivation, fast inactivation kinetics, nor recovery from inactivation of the channels (Figure 3C–E and Figure S1A; Table 2).

**Figure 4:**
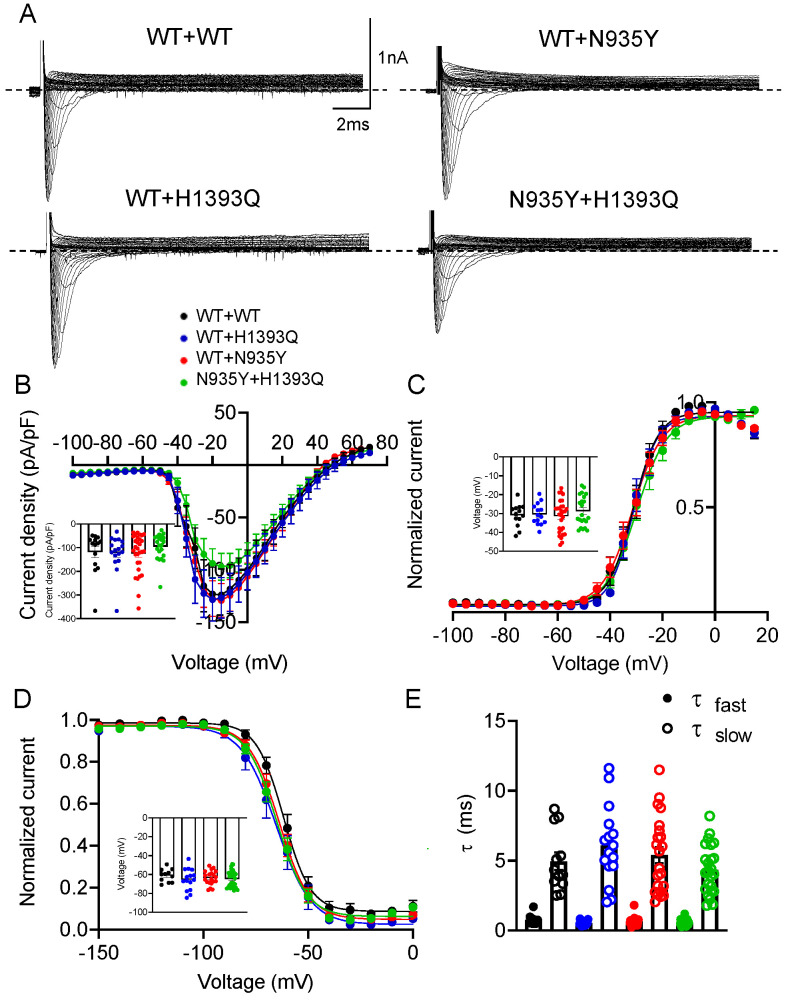
(**A**) Representative current traces evoked by 20 ms depolarizing steps from a holding potential of −150 mV to +70 mV from Nav1.1 WT + Nav1.1 WT (14 μg), Nav1.1 WT + N935Y (14 μg), Nav1.1 WT + H1393Q (14 μg) and N935Y + H1393Q (14 μg) channels expressed in HEK 293 cells. The voltage protocol is indicated in the upper panel in (**A**); (**B**) Current–voltage relationship for Nav1.1 WT + Nav1.1 WT, Nav1.1 WT + N935Y, Nav1.1 WT + H1393Q and N935Y + H1393Q channels (n = 14–22). The inset shows current density measured at −10 mV for the four channel types; (**C**) Voltage-dependent activation curves for Nav1.1 WT + Nav1.1 WT, Nav1.1 WT + N935Y, Nav1.1 WT + H1393Q and N935Y + H1393Q channels were obtained by plotting the normalized conductance as a function of the membrane potentials and fitting data points with a Boltzmann function (n = 13–29 cells). The inset shows V_1/2_ values for the four channel types; (**D**) Steady-state inactivation curves for Nav1.1 WT + Nav1.1 WT, Nav1.1 WT + N935Y, Nav1.1 WT + H1393Q and N935Y + H1393Q channels were obtained by plotting the normalized peak tail currents measured at −20 mV as a function of the prepulse potentials and fitting data points with a Boltzmann function (n = 11–27 cells). The inset shows V_1/2_ values for the four channel types; and (**E**) Bar graphs showing the time constants of the fast inactivation measured at −10 mV for the indicated channels, calculated by fitting current decay with a double exponential function (n = 12–25 cells).

Then, to model in vitro the conditions of the heterozygous mother and father, we co-transfected HEK 293 cells with an equal amount of WT and mutant cDNAs (WT + H1393Q and WT + N935Y; 7 μg + 7 μg). Similarly, to mimic the genotype of the compound heterozygous children, we co-expressed the two mutant channels (N935Y + H1393Q). All the combinations were compared with a corresponding amount of the WT cDNA condition (named WT + WT). As shown in the IV relationships in Figure 4B, the WT + H1393Q and WT + N935Y channels showed peak current densities (−121.6 ± 18.3 pA/pF and −124.4 ± 15.1 pA/pF, respectively) similar to the respective WT (118.4 ± 23.3 pA/pF) (measured at −10 mV, not statistically different). In addition, voltage-dependent activation, steady-state inactivation and fast inactivation kinetics did not show any significant difference between the groups (Figure 4C–E and Figure S1B; Table 2). This behavior could be in line with tolerated variants in the heterozygous state in the probands’ parents. The co-expression of the N935Y + H1393Q channels showed the current amplitude reduced by ~20%, albeit not significantly, with respect to WT (−95.8 ± 11.7 pA/pF vs. −118.4 ± 23.3 pA/pF) and to the paternal and maternal conditions. In addition, no difference was observed in voltage-dependence and kinetics of activation and inactivation compared with the other channels (Figure 4C–E and Figure S1B; Table 2). These data suggest a mild loss-of-function defect.

## 4. Discussion

### 4.1. Genotype–Phenotype–Drug Response Correlation

*SCN1A* variants are associated with a spectrum of neurologic disorders with variable phenotypes [27]. Variants are usually transmitted in an autosomal dominant mode or occur de novo. However, to date, 12 patients with biallelic inheritance born to unaffected heterozygous parents and blood relatives have been reported [13] (Table 1). Here, we describe two additional siblings affected by mild familial myoclonic epilepsy, cognitive delay, slurred speech, abnormal gait and hypotonia, responsive to levetiracetam, both carrying two novel missense variants in *SCN1A* inherited from asymptomatic parents.

To provide genotype–phenotype correlations for the four family members, we performed electrophysiological experiments on the H1393Q and N935Y channels, expressed alone and with a WT subunit in a heterologous expression system. We demonstrated that the H1393Q and N935Y channels cause negligible changes in Nav1.1 channel activity when expressed alone and that, in co-expression experiments, both the WT + H1393Q (maternal) and the WT + N935Y (paternal) channels share similar current density and biophysical properties with the WT protein. This functional behavior likely explains the asymptomatic condition of the probands’ parents. Regarding the previously identified recessive variants, the pathogenicity score for half of them, measured with in silico prediction tools, was low-medium [13] (Table 1). Despite functional studies having not been performed, this prediction is in line with variants that are tolerated in the heterozygous state but become pathogenic in vivo in the homozygous state [12]. Interestingly, when H1393Q and N935Y variants are inherited by the children, in a compound heterozygous state, they cause late-onset epilepsy with intellectual impairment. In this case, the functional characterization of the co-expressed N935Y + H1393Q channels demonstrated current levels slightly smaller than those of the parental channels and of the WT, with no other biophysical change. This mild loss of Nav1.1 function may explain in part the drug-responsive clinical phenotype of the probands described in this study. Indeed, as shown for other variants in *SCN1A*, besides the variant-specific effect, a more complex origin of *SCN1A* disease could be expected in patients that considers the contribution of the genetic backgrounds and neuron-type-specific homeostatic and pathologic network remodeling (for example, rare and common variants, gene regulation and modifiers) that cannot be fully reproduced in cell systems [27,28,29,30]. Defects in other epilepsy-related genes have been excluded by the genetic analysis.

In the brain, Nav1.1 channels are particularly expressed in the axons of fast-spiking inhibitory GABAergic interneurons in the neocortex, hippocampus and cerebellum [31], where channel dysfunction is expected to reduce GABA release and cause seizures and cognitive symptoms [32,33]. Though, recently, the contribution of the excitatory network has also been disclosed [34]. Thus, we could speculate that N935Y + H1393Q mild LoF would contribute to a decreased seizure threshold, thus precipitating epilepsy and co-occurring disorders.

Sodium channelopathies display varying responsiveness to drugs, principally depending on the functional defect of the underlying variants and brain network derangement. While many patients with GoF variants tend to respond to sodium channel blockers, patients with Dravet syndrome, or more in general with LoF variants, worsen with this class of drugs [2]. For example, sodium channel blockers such as carbamazepine and oxcarbazepine should be avoided in patients with Dravet syndrome, as these drugs can exacerbate the disease [35]. In our cases, sodium channel blockers such as lamotrigine and carbamazepine failed to improve the clinical outcome and were discontinued. Epilepsy was instead controlled with levetiracetam, a multitarget medication. This result supports the use of this drug in developmental and epileptic encephalopathy and would be consistent with the mild LoF identified in the functional study [36]. The involvement of glial cells and gap junction in epilepsy has been ascertained [37]. Perturbed Ca^2+^ dynamics in astrocytes [38] and glial cell activation [39] have also been reported in mouse models of Dravet syndrome. Considering that levetiracetam acts on multiple pathways [40], including astroglial gap junction coupling [41], this latter could be a possible mechanism underlying the drug effect in affected patients. 

### 4.2. Structure–Function Correlation and Comparison with Similar SCN1A Variants

The prediction of pathogenicity for a new *SCN1A* variant depends on the position on the channel structure, the type of amino acid substitution and the mode of inheritance. In general, de novo variants, truncation mutations, as well as variants affecting conserved residues in key segments for channel activity, almost always led to severe phenotypes, such as Dravet syndrome. In the case of *SCN1A* missense variants occurring at less critical channel domains, the prediction analysis, as well as the electrophysiological analysis in heterologous systems, can be more complicated [28]. As discussed above, in the case reported here, neither the H1393Q nor the N935Y inherited variants affect conserved residues across different *SCN*-genes (Figure 2). The H1393Q variant is placed in the extracellular DIIIS5-P loop and involves the substitution of a positively charged histidine by a similarly charged residue (glutamine). The N935Y is located in the DIIS5-P loop, close to a segment that stabilizes the selectivity filter and implies the replacement of asparagine with tyrosine [42]. Similarly, other variants, pathogenic only in the case of biallelic inheritance, occurred in extracellular or intracellular loops, such as the N275S mutation in the DI S5-S6 and the I923S in the DIIS5-P loop (Table 1). To date, the role of these loops in Nav1.1 activity has not been investigated in depth, but it appears that they are less critical than the VSD, the pore and the inactivation domain [42]. Despite N935 and H1393 not being conserved residues and H1393Q and N935Y behaving similarly to WT, both amino acids were already described as mutated in patients. The H1393P substitution was found in a girl presenting with remittent infantile febrile generalized tonic–clonic seizures, mild ataxia and borderline psychomotor delay [43]. In this case, the variant occurred de novo, and the first symptoms appeared at 5 months, followed by a severe phenotype. Proline is known to induce a slight kink in an alpha helix; thus, the H1393P substitution is expected to have a major impact on the channel’s structure and function, in agreement with the clinical phenotype. The mutation N935H, in which the neutral amino acid asparagine is substituted with a positively charged histidine, has been identified in a family with GEFS+ with autosomal dominant inheritance [44]. The functional study for these variants has not been performed; therefore, a genotype–phenotype correlation and a comparison with the mutants described in this study are not possible.

## 5. Conclusions

This study adds experimental evidence to previous reports of biallelic inheritance of *SCN1A* variants and suggests that some *SCN1A* variants do not cause epilepsy in the heterozygous state, but their recessive inheritance may cause it through the mild loss of Nav1.1 function. Though, other mechanisms, not easily detectable in heterologous systems, are likely involved. The integration of conventional in vitro models with patient-derived neuronal cultures will provide further insights into the molecular processes and functional alterations underlying seizures in patients with recessive inheritance of *SCN1A* variants [45].

## Figures and Tables

**Table 1:** Biallelic inheritance of *SCN1A* variants identified in this study and previously reported.

Nucleotide Change	Amino Acid Change	Protein Location	Biallelic Inheritance	N. of Patients and Phenotype	Pathogenicity Score or Functional Study	Reference
c.302G>A c.4727T>C	R101Q I1576T	Intracellular N-term DIV S2	Compound heterozygous	2, Dravet syndrome	Pathogenic Likely pathogenic	[10]
c.824A>G	N275S	DI S5-S6 extracellular loop	Homozygous	1, GEFS+	Likely pathogenic	[12]
c.1198A>G	M400V	DI S6	Homozygous	1, Dravet syndrome; and 1, febrile seizures	Pathogenic	[9]
c.1852C>T	R618C	DI-DII cytoplasmic linker	Homozygous	2, febrile seizures	Uncertain significance/Likely pathogenic	[9]
c.2768T>G	I923S	DII S5-S6 extracellular loop	Homozygous	1, GEFS+	Uncertain significance	[12]
c.2770A>T c.4146T>A	N935Y H1393Q	DII S5-S6 extracellular loop DIII S5-S6 extracellular loop	Compound heterozygous	2, developmental and epileptic encephalopathy	Likely pathogenic Likely pathogenic	This study
c.4319C>T	A1440V	DIII S5-S6 extracellular loop	Homozygous de novo	1, Dravet syndrome and acute encephalopathy	Likely pathogenic	[14]
c.4513A>C	K1505Q	DIII-DIV cytoplasmic linker	Homozygous	2, GEFS+	Likely pathogenic	[13]
c.4796delA	Y1599Ffs*19	DIV S2-S3 intracellular loop	Homozygous	1, Dravet syndrome	Pathogenic	[11]

**Table 2:** Biophysical parameters of Nav1.1 WT, H1393Q, N935Y and different combinations expressed in HEK 293 cells.

Channel Types	Current Density (−10 mV)	Voltage-Dependent Activation		Steady-State Inactivation		Kinetics of Fast Inactivation (−10 mV)		Recovery from Inactivation
	(pA/pF)	V_1/2_ (mV)	k (mV)	V_1/2_ (mV)	k (mV)	t_fast_ (ms)	t_slow_ (ms)	t (ms)
Nav1.1 WT 7 μg	−56.0 ± 9.9 (12)	−26.2 ± 3.0 (10)	3.9 ± 0.7 (10)	−59.5 ± 2.2 (11)	6.6 ± 0.8 (11)	0.72 ± 0.12 (14)	4.25 ± 0.67 (14)	2.01 ± 0.4 (6)
H1393Q 7 μg	−67.5 ± 16.9 (11)	−24.9 ± 2.2 (9)	4.7 ± 0.7 (9)	−60.8 ± 2.3 (9)	6.9 ± 0.9 (9)	0.70 ± 0.12 (14)	4.69 ± 0.57 (14)	1.77 ± 0.15 (4)
N935Y 7 μg	−64.7 ± 10.8 (10)	−25.5 ± 2.6 (9)	3.1 ± 0.5 (9)	−61.2 ± 2.4 (14)	5.3 ± 0.3 (14)	0.66 ± 0.10 (14)	5.02 ± 0.62 (14)	1.44 ± 0.25 (4)
WT + WT 7 μg + 7 μg	−118.4 ± 23.3 (14)	−30.9 ± 1.7 (13)	3.3 ± 0.4 (13)	−61.1 ± 2.1 (11)	5.0 ± 0.2 (11)	0.78 ± 0.10 (12)	4.96 ± 0.63 (12)	1.55 ± 0.13 (10)
WT + H1393Q 7 μg + 7 μg	−121.6 ± 18.3 (17)	−30.4 ± 1.5 (14)	3.2 ± 0.4 (14)	−65.3 ± 2.9 (15)	5.02 ± 0.2 (15)	0.54 ± 0.04 (16)	6.08 ± 0.69 (16)	1.33 ± 0.15 (9)
WT + N935Y 7 μg + 7 μg	−124.4 ± 15.1 (18)	−31.3 ± 1.6 (29)	3.5 ± 0.2 (29)	−63.6 ± 1.7 (20)	5.8 ± 0.3 (20)	0.66 ± 0.06 (22)	5.4 ± 0.58 (22)	2.06 ± 0.49 (8)
N935Y + H1393Q 7 μg + 7 μg	−95.8 ± 11.7 (22)	−28.7 ± 1.7 (22)	4.0 ± 0.4 (22)	−64.7 ± 1.7 (27)	−5.3 ± 0.2 (27)	0.60 ± 0.04 (25)	4.36 ± 0.34 (25)	2.71 ± 0.56 (8)

Data are mean ± SE. The number of cells is indicated in round brackets.

## Data Availability

Genetic data are reported in the Leiden Open Variation Databases (LOVD) https://databases.lovd.nl/shared/variants/SCN1A/unique#object_id=VariantOnTranscriptUnique%2CVariantOnGenome&id=SCN1A&search_transcriptid=00023858&search_owned_by_=Palumbo&page_size=100&page=1, accessed on 2 June 2023.

## Supplementary Materials

The following supporting information can be downloaded at: https://www.mdpi.com/article/10.3390/biomedicines12081698/s1, Figure S1: Recovery from inactivation.
