# Supplementary material for: The Biallelic Inheritance of Two Novel SCN1A Variants Results in Developmental and Epileptic Encephalopathy Responsive to Levetiracetam
Source: Biomedicines. 2024 Jul 31;12(8):1698. doi: 10.3390/biomedicines12081698 (PMC11351414; doi:10.3390/biomedicines12081698)
Supplement: Supplementary file 1 [file biomedicines-12-01698-s001.zip › biomedicines-3115963-supplementary.pdf]

biomedicines

MDPI

Supplementary File

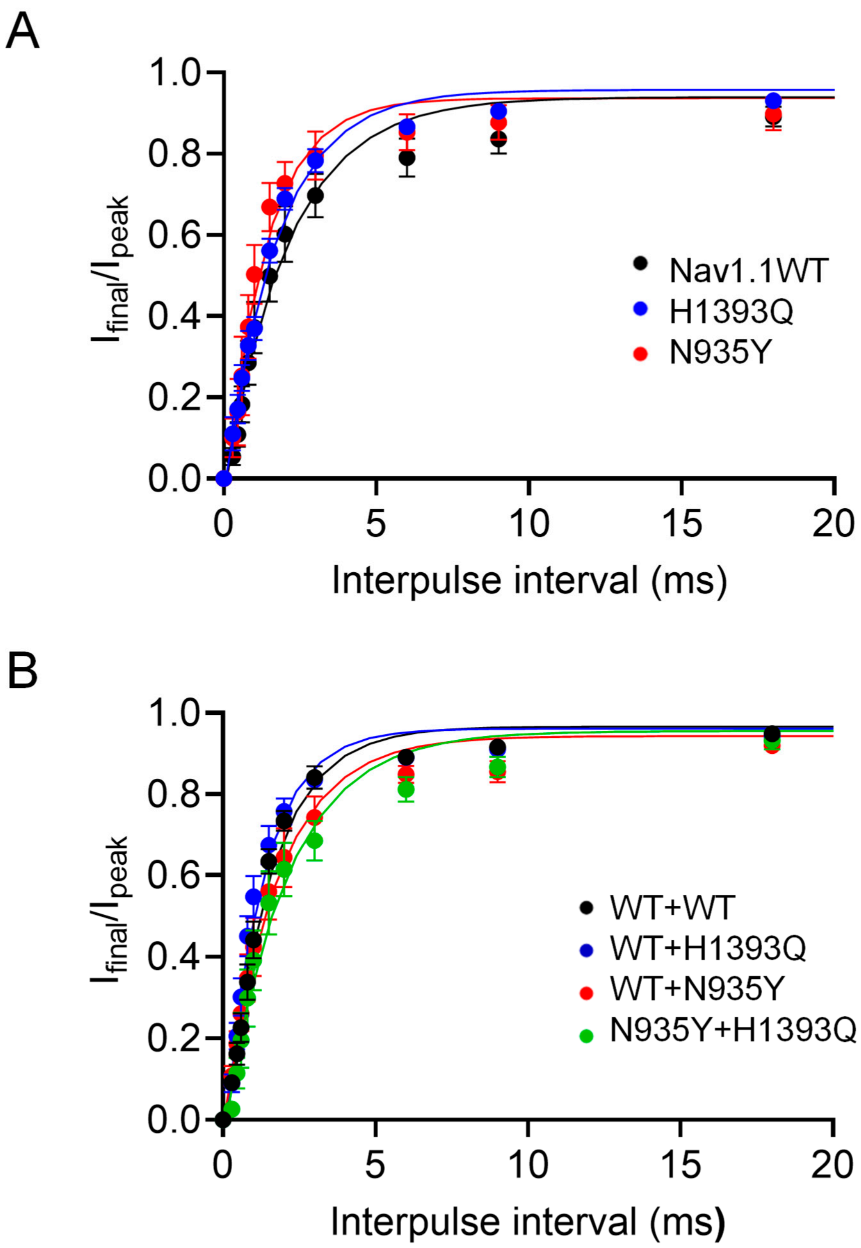

Figure S1. (**A**) Recovery from inactivation of Nav1.1WT (7 µg), N935Y (7 µg), and H1393Q (7 µg) channels expressed in HEK 293 cells. (**B**) Recovery from inactivation of Nav1.1WT+Nav1.1WT (14 µg), Nav1.1WT+N935Y (14 µg), Nav1.1WT+H1393Q (14 µg) and N935Y+H1393Q (14 µg) channels expressed in HEK 293 cells.
